# Pain Is Widespread and Predicts Poor Mental Health Among Older Adults in Rural Malawi

**DOI:** 10.1093/geroni/igac008

**Published:** 2022-03-05

**Authors:** Iliana V Kohler, Alberto Ciancio, Fabrice Kämpfen, Hans-Peter Kohler, Victor Mwapasa, Benson Chilima, Steve Vinkhumbo, James Mwera, Jürgen Maurer

**Affiliations:** Population Studies Center, University of Pennsylvania, Philadelphia, Pennsylvania, USA; Department of Sociology, University of Pennsylvania, Philadelphia, Pennsylvania, USA; Adam Smith Business School, University of Glasgow, Glasgow, UK; School of Economics, University College Dublin, Dublin, Ireland; Department of Sociology, University of Pennsylvania, Philadelphia, Pennsylvania, USA; Population Aging Research Center, University of Pennsylvania, Philadelphia, Pennsylvania, USA; Kamuzu University of Health Sciences, Blantyre, Malawi; Ministry of Health, Lilongwe, Malawi; Ministry of Gender, Children, Disability and Social Welfare, Lilongwe, Malawi; Invest in Knowledge Initiative, Zomba, Malawi; Department of Economics, University of Lausanne, Lausanne, Switzerland

**Keywords:** Depression, Gender differences, PHQ-9, Physical health, Sub-Saharan Africa

## Abstract

**Background and Objectives:**

Pain is common among older persons and has been documented as an important predictor of disability, health, and economic outcomes. Evidence about its prevalence and relationship to well-being is scarce in rural sub-Saharan Africa (SSA), where work is frequently physically demanding, and pain prevention or treatment options are limited. We investigate the prevalence of pain and its association with mental health and subjective well-being in a population-based study of older adults in rural Malawi.

**Research Design and Methods:**

We estimate the prevalence, severity, and duration of pain along with its sociodemographic distribution in a sample of 1,577 individuals aged 45 and older. We assess the association of pain with clinically validated measures of mental health, including depression and anxiety, and subjective well-being.

**Results:**

Pain is widespread in this mature population with an average age of 60 years: 62% of respondents report the experience of at least minor pain during the last year, and half of these cases report severe or disabling pain. Women are more likely to report pain than men. Pain is a strong predictor of mental health and subjective well-being for both genders. More severe or longer pain episodes are associated with worse mental states. Individuals reporting pain are more likely to suffer from depression or express suicidal thoughts.

**Discussion and Implications:**

Our study identifies key subpopulations such as older women in a SSA low-income context who are particularly affected by the experience of pain in daily life and calls for interventions targeting pain and its consequences for mental health and subjective well-being.

**Translational Significance**

Our results emphasize the importance of prioritizing pain and mental health among older individuals in sub-Sahara African (SSA) low-income countries, to which relatively few health care resources are currently allocated. Expanding information about and access to effective pain treatments in nonclinical settings in SSA can potentially lead to significant increases in population health. Focus needs to be on nonclinical populations as pain is widespread among older adults who do not frequently interact with the health system. Pain may be an important contributor to poor mental health. Reducing pain should be considered an integral part of addressing the poor mental health among older adults.

The increasing prevalence of pain constitutes one of the most common and costly health burdens worldwide (GBD 2015 Disease and Injury Incidence and Prevalence Collaborators, 2016). Although pain is frequently associated with a disease or injury, it represents on its own a disabling health condition with profound implications for people’s quality of life (QOL) (Mills et al., 2019). While pain and its implications for QOL have been extensively studied in high- or middle-income contexts, evidence about prevalence, disparities, and QOL implications of pain remains scarce in low-income countries (LICs), where adult and older populations are disproportionally exposed to risk factors for developing pain such as demanding physical work combined with frequent undernutrition, and access to effective pain treatments or management options is often limited.

Chronic musculoskeletal pain such as low back pain or neck pain currently represents the leading cause of disability in many countries, and between 2005 and 2015 the global years lived with disability (YLD) caused by both types of pain increased by 18.6% (GBD 2015 Disease and Injury Incidence and Prevalence Collaborators, 2016; Hurwitz et al., 2018). This global burden of pain is projected to further increase in the next decades, in part as a result of population aging, and especially so in low- and middle-income countries. The health, social, and economic implications of pain are increasingly affecting populations across the entire development spectrum (Buchbinder et al., 2018; Chatterji et al., 2015; GBD 2017 Disease and Injury Incidence and Prevalence Collaborators, 2018; Murray et al., 2012; Velkoff & Kowal, 2006). For instance, of the 46 countries in the sub-Saharan African (SSA) region, nine had lower back and neck pain as the leading cause of YLD in 2015 (GBD 2015 Disease and Injury Incidence and Prevalence Collaborators, 2016).

Pain has a large adverse impact on individuals’ lives. Activity limitations, reduced work capacity, and restricted social participation frequently result directly from pain (Louw, 2007). Poor mental health and/or depression often represent direct or indirect consequences of pain, particularly in contexts where treatment or management for pain is insufficient (Stubbs et al., 2016; Tsang et al., 2008). Chronic pain and mental health disorders are thus often co-occurring—a pattern that is well-documented in high- and middle-income countries, but possibly is even more important in LICs where pain affects already vulnerable individuals whose livelihood often depends on physical activities. Combined, pain and poor mental health already represent the globally leading causes of disease and disability (GBD 2017 Disease and Injury Incidence and Prevalence Collaborators, 2018), and they constitute an important impediment in LICs to increases in health, productivity, well-being, and overall socioeconomic development (Case et al., 2020).

The treatment and management of pain are increasingly seen as central aspects in health policy debates and health care guidelines (National Academies of Sciences, Engineering, and Medicine, 2017; Morriss and Roques 2018; Vadivelu et al., 2018). In SSA, however, most of the existing evidence on the prevalence and correlates of pain is based on two countries, South Africa and Nigeria, that took part in the World Mental Health Surveys (Demyttenaere, 2007). Both of these countries represent lower- and upper-middle-income countries and corresponding findings may not generalize to SSA LICs, where pain has not been extensively investigated. One exception is a study by Stubbs (2016) that, utilizing data from the World Health Survey of 2002–2004, expanded the geographic coverage of research on pain and mental well-being by including several SSA LICs. These studies, however, do not specifically document pain and its QOL implications among older adults who might be most affected by pain as a result of sustained exposures to life course adversities, poverty, and other risk factors for pain (e.g., the mean age covered in the study by Stubbs is 38 years).

We contribute to current research on pain in LICs by investigating the prevalence, severity, and duration of pain along with its sociodemographic distribution in a sample of 1,577 older individuals older than age 45 years living in rural Malawi, a low-income SSA country that is ranked 172 out of 189 countries on the Human Development Index (United Nations Development Programme, 2019). Similar to other low- and middle-income countries, the longer-run disease burden in Malawi is shifting from infectious diseases to noncommunicable diseases (NCDs), with the latter becoming major causes of morbidity and mortality. Besides highlighting the prevalence of pain and its sociodemographic distribution, we also describe the association of pain with several measures of mental health and subjective well-being, including Short Form Survey (SF-12) mental health score, depression (patient health questionnaire-9 [PHQ-9]), and anxiety (generalized anxiety disorder-7 [GAD-7]), to assess in part its impact on people’s everyday life. To the best of our knowledge, we provide one of the first studies that documents the prevalence of pain in an aging nonclinical SSA LICs population and analyzes in this older population the association of pain prevalence, severity, and duration with poor mental health and subjective well-being.

## Method

### Study Population

Our analyses are based on the Mature Adults Cohort of the Malawi Longitudinal Study of Family and Health (MLSFH-MAC) that collected in 2017 comprehensive data on pain and other dimensions of health in older adults in rural Malawi (Kohler et al., 2020). The MLSFH-MAC is a population-based cohort study of mature adults aged 45 years and older who live overwhelmingly in rural communities in three districts in Malawi (Mchinji in the central, Rumphi in the northern, and Balaka in the southern region). The MLSFH-MAC was established in 2012 by selecting respondents from the MLSFH (Kohler et al., 2015), currently with follow-up waves in 2013, 2017, and 2018. The original MLSFH sample in 1998 was based on a probabilistic population sample, with the sample being augmented by enrolling adolescents, parents, and new spouses of respondents in the later rounds of data collections (for details, see Kohler et al., 2015). Comparisons of the 2010 MLSFH study population with the rural samples of the Malawi DHS and Integrated Household Survey 3 (IHS3) surveys reveal that the study closely matches the rural subsample in the 2010 nationally representative IHS3 in key observable characteristics (Kohler et al., 2020, 2015; see Author Notes 1 and 2). Detailed information on the MLSFH-MAC sampling procedures, comparisons of the study population with nationally representative samples, study design, and study instruments are provided in the MLSFH-MAC Cohort Profile (Kohler et al., 2020).

The MLSFH-MAC includes extensive information on physical, mental, and cognitive health, NCDs-related health knowledge and health care utilization, socioeconomic well-being, household production and consumption, household structure, intergenerational transfers, and family relationships. While not nationally representative, the MLSFH-MAC broadly represents older persons living in rural Malawi, where 85% of all Malawians live (National Statistical Office, 2019). Most of the individuals living in these rural areas engage in manual, intensive physical labor such as home production of crops, complemented by some market activities.

The present analyses are cross-sectional and utilize the MLSFH-MAC data from 2017 when detailed information on pain was collected for the first time. The target sample was 1,814 respondents, of whom 88.5% were successfully interviewed. The main reason for not participating in the study was because of mortality (8% of respondents died between 2013 and 2017). Only nine respondents refused to participate in the survey (0.5%). The others were either temporarily absent, not found, or migrated outside of the study areas without sufficient details for follow-up.

Our analytical sample includes MLSFH-MAC respondents with nonmissing information in the questions eliciting the experience of pain and in basic demographic characteristics including age, gender, schooling, and wealth (see Author Note 3). The final sample consists of 1,577 respondents who were 45 years or older in 2017 (Table 1). Mean age of the study sample was 59.5 years, and about 40% of the sample were men. Respondents had low levels of schooling (mean years of schooling was 3.57 years), with men having attained higher levels of education (mean = 4.74 years) compared to women (mean = 2.79). Among men, about 94% were married at the time of the survey versus only 58.8% of women. Marriage is almost universal in Malawi, and this gender difference in marital status stems from a higher proportion of widowed women. Respondents were about equally distributed across the three MLSFH study regions.

**Table 1.** Summary Statistics

	All		Women		Men		Difference
Variable	Mean	*SD*	Mean	*SD*	Mean	*SD*	*p*
**Panel A: Individual characteristics**							
Male	0.398	0.490					
Age	59.499	11.833	59.171	11.983	59.997	11.595	.175
Years of schooling	3.569	3.433	2.794	3.084	4.743	3.600	.000
Wealth							
Second tertile	0.386	0.487	0.416	0.493	0.341	0.475	.003
Third tertile	0.258	0.438	0.198	0.399	0.349	0.477	.000
Married	0.726	0.446	0.588	0.492	0.935	0.247	.000
Balaka	0.344	0.475	0.368	0.482	0.308	0.462	.015
Mchinji	0.327	0.469	0.300	0.459	0.367	0.482	.005
Rumphi	0.329	0.470	0.332	0.471	0.324	0.468	.752
**Panel B: Pain variables**							
No lasting pain	0.380	0.485	0.337	0.473	0.445	0.497	.000
Slight	0.129	0.336	0.144	0.351	0.107	0.309	.031
Moderate	0.181	0.385	0.174	0.379	0.193	0.395	.331
Severe	0.113	0.317	0.118	0.323	0.105	0.307	.438
Disabling	0.197	0.398	0.227	0.419	0.150	0.357	.000
Duration							
<1 month	0.528	0.499	0.568	0.496	0.468	0.499	.000
2–3 months	0.060	0.238	0.059	0.236	0.062	0.242	.793
3+ months	0.031	0.174	0.036	0.186	0.024	0.153	.183
**Panel C: Pain variables conditional on pain**							
Slight	0.209	0.407	0.217	0.413	0.193	0.395	.359
Moderate	0.292	0.455	0.262	0.440	0.348	0.477	.005
Severe	0.182	0.386	0.178	0.383	0.190	0.393	.645
Disabling	0.317	0.466	0.343	0.475	0.270	0.445	.019
Duration							
<1 month	0.852	0.355	0.857	0.351	0.844	0.363	.604
2–3 months	0.097	0.297	0.089	0.285	0.112	0.316	.242
3+ months	0.050	0.219	0.054	0.226	0.043	0.204	.456
Any treatment	0.946	0.226	0.946	0.226	0.945	0.228	.967
**Panel D: Mental health and well-being**							
SF-12 mental health	49.481	10.367	48.481	10.652	51.003	9.730	.000
PHQ-9 depression score	4.247	4.281	4.655	4.408	3.630	4.006	.000
GAD-7 anxiety score	3.552	3.467	3.951	3.589	2.947	3.182	.000
Subjective well-being	3.637	1.026	3.561	0.998	3.751	1.057	.000
Depression level							
At least mild	0.394	0.489	0.435	0.496	0.332	0.471	.000
At least moderate	0.104	0.305	0.117	0.321	0.085	0.278	.040
Suicidal/self-harming thoughts	0.073	0.260	0.093	0.290	0.043	0.203	.000
Observations	1,577		950		627		1,577

*Notes:* Summary statistics for the whole sample and by gender. The last column indicates the *p* value from a two-sample *t* test of the difference between men and women means. No lasting pain indicates no pain episodes that lasted at least 1 week in the past year.

### Assessment of Pain

Self-reported pain among MLSFH-MAC respondents was assessed with the question on pain implemented in the Health and Retirement Study (HRS) Experimental Module in 2010: “During the past year, have you experienced pain that lasted for one week or longer?” To reduce the time required for the implementation of the survey, the response options were expanded to also measure severity of pain. Specifically, MLSFH-MAC respondents were asked “During the past year, have you experienced pain that lasted for one week or longer?”, with interviewers being instructed to read the response categories “Yes, disabling pain,” “Yes, severe pain,” “Yes, modest pain,” “Yes, slight pain,” and “No pain.” Responses to the question on pain therefore indicate the presence or absence of pain, as well as the severity of pain (conditional of experiencing pain).

If respondents reported a pain episode that lasted more than 1 week, they were asked about the duration of the most severe episode of pain during the past year implementing the same categorization as in the HRS module on pain. Based on this information, we constructed four categories for the pain duration: no pain episode lasting for 1 week or longer, pain lasting less than a month, pain lasting 2–3 months, and pain lasting more than 3 months. Pain with a duration of more than 3 months is recognized as chronic pain (Treede et al., 2015). The survey also asked whether respondents used any treatment for the most severe episode of pain assessed with the previous questions that lasted more than 1 week.

### Assessment of Mental Health and Subjective Well-Being

MLSFH-MAC collected detailed information on several dimensions of mental health and subjective well-being. Depression was elicited by the PHQ-9 instrument, that is, a clinically validated measurement of the presence and severity of depression. The PHQ-9 score is based on nine questions that ask the respondents how often they have been bothered in the past 2 weeks before the survey by the following such as “little interest or pleasure in doing things”; “feeling down, depressed, or hopeless”; “feeling bad about yourself—or that you are a failure or have let yourself or your family down.” Response categories for all questions in the PHQ-9 range from 0 (*not at all*) to 3 (*nearly every day*). The overall PHQ-9 score ranging between 0 and 27 corresponds to the sum of the values of all individual questions in this instrument. Official guidelines consider a PHQ-9 score between 5 and 9 as mild depression and a score above 9 as at least moderate depression (Kroenke & Spitzer, 2010; Kroenke et al., 2002). If a respondent gave any response other than “not at all” to the final PHQ-9 question about “thoughts that you would be better off dead or of hurting yourself in some way,” he/she was classified as having self-harming or suicidal thoughts. MLSFH-MAC also implemented the GAD-7 instrument that indicates the presence and severity of anxiety with a range from 0 to 21 (from least to worst anxiety). GAD-7 includes seven questions that ask the respondent to categorize if or how often they have been bothered in the past 4 weeks by the experience of the following such as: “feeling nervous, anxious, or on edge”; “not being able to stop or control worrying”; or “feeling afraid as if something awful might happen. Similarly, to PHQ-9, the GAD-7 individual response categories range from 0 (*not at all*) to 3 (*nearly every day*). The GAD7 score is the sum of all values of individual GAD-7 questions. The guidelines for the GAD-7 measure specify scores of 5, 10, and 15 as cut points for mild, moderate, and severe anxiety (Kroenke & Spitzer, 2010). PHQ-9 and GAD-7 have been shown to be reliable and valid instruments to measure depression and anxiety in different income contexts, including SSA LICs and specifically Malawi (Brinkmann et al., 2020; Kessler & Bromet, 2013; Kohler et al., 2017; Sweetland et al., 2014). In addition, MLSFH-MAC collects the 12-item Short Form Survey (SF-12) instrument, which provides a widely utilized measure of overall mental health. The SF-12 has been validated in many contexts (Gandek et al., 1998), including Malawi (Ohrenberger et al., 2020). The survey also elicits information on overall subjective well-being via the commonly used question: “I am interested in your general level of well-being or satisfaction with life. How satisfied are you with your life, all things considered?” with responses being measured using a 5-point Likert scale ranging from “very unsatisfied” to “very satisfied.”

### Explanatory Variables

Explanatory variables included in our analyses cover demographic and socioeconomic characteristics that are commonly predictive of health outcomes among older individuals such as age, gender, schooling, wealth, marital status, and region of residence. Age was segmented into 10-year groups (below 50 years, 50–59 years, 60–69 years, and 69+ years). To measure schooling attainment of respondents, we used years of schooling that range from 0 to 12+ years. Wealth is measured via a 14-item score that accounts for the household ownership of assets such as a metal roof, a radio, or a phone (Chin, 2010). In the analysis, we included binary variables for each tertile of the overall wealth index. Finally, all our models control for the MLSFH-MAC study regions (Balaka, Mchinji, and Rumphi) to account for cultural, social, and economic differences across these geographic areas.

### Statistical Analysis

We first analyze the relationship between pain and individual characteristics by estimating probit and ordered probit regression models. Probit regressions are used for binary outcomes such as whether a respondent reports the experience of any pain. Ordered probit regressions are used in analyses of ordered outcomes such as the severity of pain measured by a categorical variable ranging from 0 “no pain” (lowest category) to “slight,” “moderate,” “severe,” and “disabling pain” (highest category). To analyze the relationship between pain and mental health, we use ordinary least squares regressions for the outcomes SF-12 mental health score, the PHQ-9 total depression score, the GAD-7 total anxiety score, and subjective well-being (i.e., overall life satisfaction); probit regressions are used for binary outcomes that measure the presence of mild depression, moderate depression, or having suicidal or self-harming thoughts. To facilitate comparability of effect sizes and ease of interpretation, all continuous mental health measures are standardized to mean zero and standard deviation equal to 1. All analyses were performed using Stata 14.1 (STATA Corp LP, College Station, TX).

### Ethics Approval

The data collections of the MLSFH-MAC and MLSFH have been approved by the Internal Review Board (IRB) at the University of Pennsylvania (IRB Protocols #815016 and #826828). In Malawi, the MLSFH-MAC and MLSFH research has been approved by the Ethics Committee of the College of Medicine, Malawi (COMREC, Protocols #P01/12/1165 and #P04/17/2160) and the National Health Sciences Research Committee (NHSRC, Protocol #19/01/2214).

## Results

### Prevalence, Severity, and Duration of Pain

**Figure 1.**
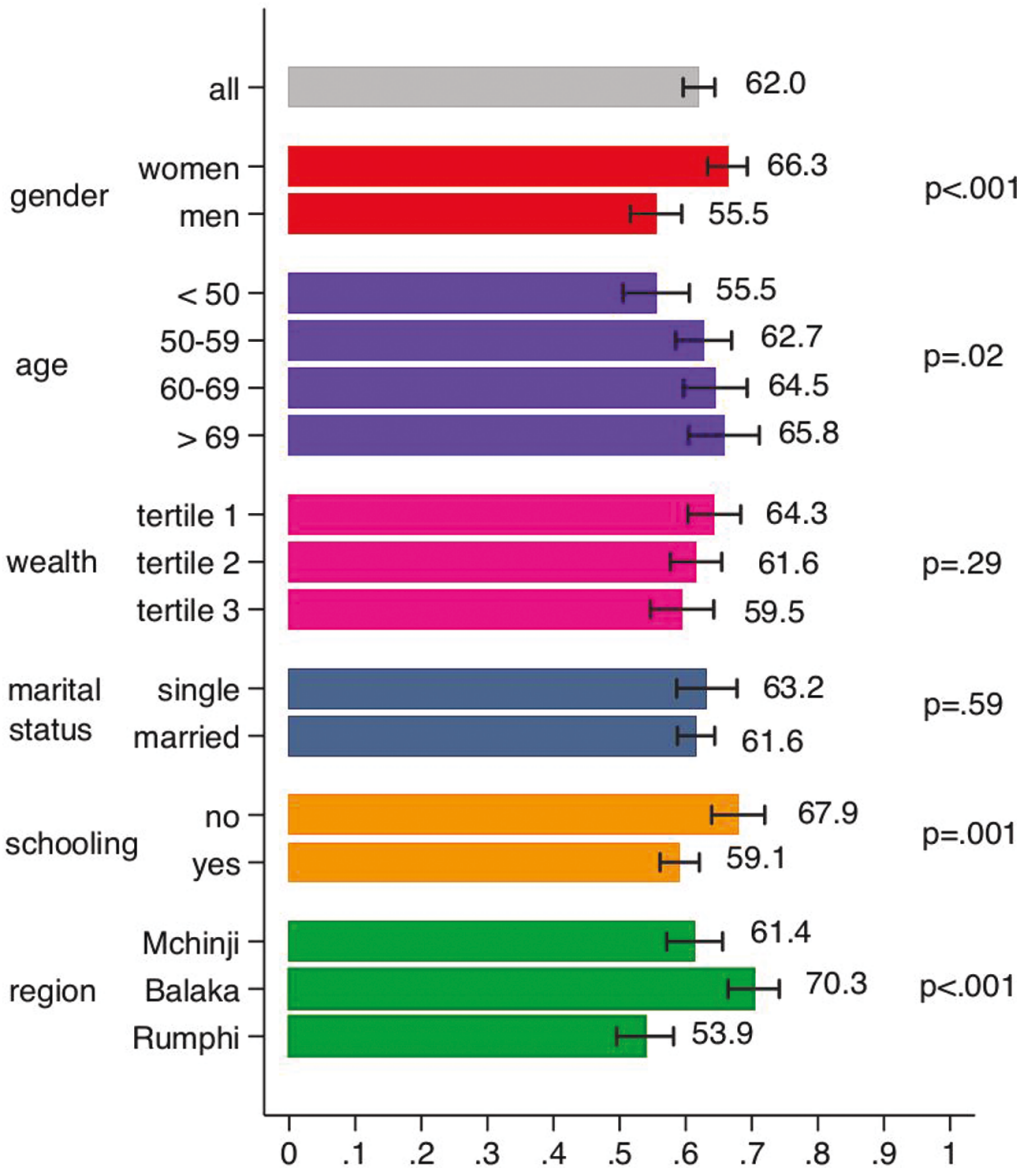
Prevalence of self-reported pain in the overall sample and results of descriptive unadjusted bivariate analyses for selected sociodemographic characteristics.

Figure 1 shows the prevalence of self-reported pain in the overall sample and the results of descriptive unadjusted bivariate analyses for selected sociodemographic characteristics. About 62% of the MLSFH-MAC respondents report the experience of at least mild pain that lasted a week or longer during the past year. There are also important gender differences in pain reports, with women more likely to report any pain (66.3% vs. 55.5% of men). This difference is statistically significant (*z*-test for equality of proportions = 4.33). We also observe a positive age gradient in the experience of pain, with older persons more frequently reporting pain than younger persons. Pain prevalence is also higher among individuals with no schooling, and we observe significant differences in pain prevalence by region (highest prevalence occurs in the southern region Balaka).

The unadjusted distribution of the severity of pain is shown in Panel B of Table 1. Only 38% of respondents reported no pain, and 13% reported slight pain. Of the respondents, 49% experienced *at least* moderate pain in the past year (18% moderate, 11% severe, and 20% disabling pain), corresponding to 79% of all respondents who reported any pain episode (Panel C). About 31% of the respondents experienced at least severe pain. About 9% of respondents reported that the most severe experience of pain lasted more than a month. Conditional on reporting any pain, 32% of respondents experienced disabling pain, 15% of the respondents reported that their most severe pain episode lasted more than a month, and 5% reported pain with a 3+ months duration. Gender differences in pain are also important: 22.7% of women suffered from disabling pain versus only 15% of men. Women were also slightly more likely to experience pain that lasts for more than a month (9.5% of women vs. 8.6% of men), but the difference is not statistically significant. About 95% of respondents whose most severe pain episode over the last year lasted 1 week or longer reported using some treatment to address their pain (Panel C in Table 1).

### Associations of Pain With Individual Characteristics

Table 2 displays the partial association of pain with mature adults’ sociodemographic characteristics. The multivariate estimates in Column 1 indicate the marginal effects of a probit regression of having any lasting pain on demographic characteristics. Consistent with Figure 1, these multivariate analyses also document that men are less likely to experience pain than women; the likelihood of experiencing pain is also positively correlated with age and being married and negatively correlated with schooling. The age gradient is steep from ages 45 to 60–69 years while it is flatter for older ages (from 60–69 to 70+ years). There are also significant differences in reported pain by region: Older adults residing in the southern region (Balaka) reported more pain than in the central region (Mchinji, omitted) and in the northern region (Rumphi).

**Table 2.** Models Showing Associations Between Pain and Individual Characteristics

	Any lasting pain	Severity					Duration			
	Regressors	No lasting pain	Mild	Moderate	Severe	Disabling	No lasting pain	<1 month	2–3 months	>3 months
	(1)	(2)	(3)	(4)	(5)	(6)	(7)	(8)	(9)	(10)
Male	−0.115** (0.028)	0.117** (0.024)	0.005** (0.002)	−0.014** (0.003)	−0.023** (0.005)	−0.085** (0.018)	0.087** (0.025)	−0.049** (0.014)	−0.021** (0.006)	−0.016** (0.005)
Age 50–59	0.072* (0.033)	−0.033 (0.029)	−0.001 (0.001)	0.005 (0.004)	0.007 (0.006)	0.022 (0.019)	−0.070* (0.030)	0.044* (0.020)	0.015* (0.007)	0.010* (0.005)
Age 60–69	0.101** (0.035)	−0.073* (0.031)	−0.003† (0.002)	0.010* (0.004)	0.014* (0.006)	0.052* (0.022)	−0.093** (0.032)	0.057** (0.020)	0.021** (0.008)	0.015** (0.006)
Age 70+	0.110** (0.039)	−0.083* (0.034)	−0.004† (0.002)	0.010* (0.004)	0.016* (0.007)	0.060* (0.025)	−0.138** (0.035)	0.079** (0.020)	0.034** (0.009)	0.025** (0.007)
Years of schooling	−0.008† (0.005)	0.003 (0.004)	0.000 (0.000)	−0.000 (0.001)	−0.001 (0.001)	−0.002 (0.003)	0.008† (0.004)	−0.004† (0.003)	−0.002† (0.001)	−0.001† (0.001)
Wealth second tertile	0.023 (0.030)	0.012 (0.025)	0.001 (0.001)	−0.001 (0.003)	−0.002 (0.005)	−0.009 (0.018)	−0.023 (0.026)	0.013 (0.015)	0.006 (0.007)	0.004 (0.005)
Wealth third tertile	0.056 (0.036)	−0.025 (0.031)	−0.001 (0.001)	0.003 (0.004)	0.005 (0.006)	0.018 (0.023)	−0.042 (0.032)	0.024 (0.018)	0.010 (0.008)	0.008 (0.006)
Married	0.056† (0.031)	−0.052† (0.027)	−0.002† (0.001)	0.006† (0.003)	0.010† (0.005)	0.038† (0.020)	−0.035 (0.028)	0.020 (0.016)	0.009 (0.007)	0.007 (0.005)
Balaka	0.067* (0.032)	−0.021 (0.027)	−0.001 (0.002)	0.002 (0.003)	0.004 (0.005)	0.016 (0.021)	−0.051† (0.028)	0.027† (0.015)	0.013† (0.008)	0.011† (0.006)
Rumphi	−0.070* (0.033)	0.071* (0.029)	0.002† (0.001)	−0.010* (0.004)	−0.014* (0.006)	−0.049* (0.020)	0.053† (0.030)	−0.033† (0.019)	−0.012† (0.007)	−0.008† (0.005)

*Notes:* The table shows average marginal effects from probit regression models (any pain) or ordered probit regressions models (severity and duration). No lasting pain indicates no pain episodes that lasted at least 1 week in the past year. Severity of pain is a categorical variable with five groups from no lasting pain to disabling pain. Duration of pain is a categorical variable with four groups from no lasting pain to pain that lasts more than 3 months. The reference categories are female, age <50 years, first tertile of the wealth distribution, single marital status (includes single, divorced, and widowed), and residing in Mchinji.

†*p* < .1, *p < .05, **p < .01.

Columns 2–6 of Table 2 display the average marginal effects of an ordered probit regression for pain severity. Results are qualitatively similar to the results for reporting any pain. We see a positive partial correlation between pain severity and being a woman, age, being married, and not being from the northern region. For example, women were 8.5 percentage points (pp) more likely to report disabling pain than men. Similarly, individuals aged 70 years or older were 6 pp more likely to report disabling pain when controlling for other characteristics. The average marginal effect for schooling is negative but not statistically significant.

Columns 7–10 of Table 2 display the average marginal effects of an analogous ordered probit regression for pain duration. Other things equal, male mature adults are more likely to have short episodes of pain than female mature adults. Episodes of pain experienced by younger mature adults tend to be longer than those experienced by older individuals. For example, men are 4.9 pp less likely than women to experience pain for less than a month but only 1.6 pp less likely to experience pain that lasts more than 3 months. Similarly, respondents 70 years or older are 7.9 pp more likely to experience short episodes of pain but only 2.5 pp more likely to experience episodes of pain longer than 3 months. There is no significant association between pain duration and wealth or marital status.

### Associations Between Pain and Mental Health

Overall, 39% of MLSFH mature adults had at least mild depression and 10% at least moderate depression, with women having worse mental health and being more likely to suffer from depression and anxiety (Table 1). About 7% of MLSFH-MAC study participants reported self-harming thoughts during the last 2 weeks before the survey, with women significantly more like to do so compared to men (9% vs. 4% among men; *p* < .001).

Table 3 displays our estimation results for the partial associations between pain and subjective well-being and different measures of mental health, respectively. We see a clear negative relationship between the SF-12 mental health score and pain. The relationship is monotonic in pain severity with the association becoming stronger when the episode of pain is more severe. Similarly, we find a positive association of pain severity with the PHQ-9 depression score and the GAD-7 anxiety score and a negative association with subjective well-being. In Table 3, we also estimate the association between mental health and pain duration. Again, we see a clear monotonic negative relationship between pain duration and the SF-12 score and subjective well-being and a positive monotonic relationship with depression and anxiety. For instance, individuals who experienced an episode of pain that lasted more than 3 months have a depression score that is 1 *SD* larger than others. Specifications with pain severity and duration together show that both dimensions are significant predictors of mental health. Results by gender show a very similar pattern (Supplementary Tables 1–4).

**Table 3.** Models Showing Association Between Mental Health and Well-Being With Pain Severity and Duration

	SF-12 mental health			PHQ-9 depression score			GAD-7 anxiety score			Subjective well-being		
Regressors	(1)	(2)	(3)	(4)	(5)	(6)	(7)	(8)	(9)	(10)	(11)	(12)
Slight	−0.147† (0.079)		−0.143† (0.079)	0.133† (0.077)		0.129† (0.077)	0.134† (0.077)		0.130† (0.077)	−0.005 (0.080)		−0.004 (0.080)
Moderate	−0.268** (0.069)		−0.254** (0.070)	0.316** (0.068)		0.291** (0.068)	0.296** (0.067)		0.274** (0.068)	−0.210** (0.070)		−0.199** (0.071)
Severe	−0.378** (0.084)		−0.312** (0.087)	0.517** (0.082)		0.446** (0.085)	0.479** (0.081)		0.405** (0.084)	−0.191* (0.084)		−0.161† (0.088)
Disabling	−0.426** (0.068)		−0.373** (0.070)	0.536** (0.066)		0.468** (0.069)	0.538** (0.066)		0.471** (0.068)	−0.190** (0.069)		−0.161* (0.071)
Duration <1 month		−0.272** (0.052)			0.329** (0.051)			0.318** (0.051)			−0.137** (0.053)	
Duration 2–3 months		−0.488** (0.106)	−0.167 (0.106)		0.559** (0.104)	0.144 (0.104)		0.541** (0.103)	0.144 (0.103)		−0.168 (0.108)	−0.003 (0.108)
Duration >3 months		−0.666** (0.143)	−0.327* (0.143)		0.957** (0.140)	0.525** (0.140)		0.934** (0.139)	0.510** (0.139)		−0.467** (0.145)	−0.301* (0.145)
Male	0.176** (0.058)	0.185** (0.057)	0.178** (0.058)	−0.174** (0.056)	−0.184** (0.056)	−0.174** (0.056)	−0.209** (0.056)	−0.220** (0.056)	−0.208** (0.056)	0.105† (0.058)	0.102† (0.058)	0.103† (0.058)
Age 50–59	−0.074 (0.066)	−0.062 (0.066)	−0.069 (0.066)	0.011 (0.064)	−0.009 (0.064)	0.002 (0.064)	0.022 (0.064)	0.002 (0.064)	0.014 (0.064)	−0.175** (0.066)	−0.169* (0.066)	−0.170* (0.066)
Age 60–69	−0.220** (0.071)	−0.216** (0.071)	−0.219** (0.071)	0.239** (0.070)	0.225** (0.070)	0.231** (0.069)	0.254** (0.069)	0.242** (0.069)	0.247** (0.069)	−0.182* (0.072)	−0.176* (0.072)	−0.180* (0.072)
Age 70+	−0.393** (0.079)	−0.368** (0.079)	−0.372** (0.079)	0.543** (0.077)	0.508** (0.077)	0.515** (0.077)	0.625** (0.077)	0.590** (0.077)	0.597** (0.076)	−0.481** (0.080)	−0.457** (0.080)	−0.462** (0.080)
Years of schooling	−0.013 (0.010)	−0.015 (0.010)	−0.013 (0.010)	0.008 (0.010)	0.009 (0.010)	0.007 (0.010)	0.008 (0.010)	0.009 (0.010)	0.007 (0.010)	−0.001 (0.010)	−0.001 (0.010)	−0.000 (0.010)
Wealth second tertile	0.173** (0.060)	0.191** (0.059)	0.179** (0.060)	−0.110† (0.058)	−0.138* (0.058)	−0.119* (0.058)	−0.020 (0.058)	−0.047 (0.058)	−0.029 (0.058)	0.057 (0.060)	0.074 (0.060)	0.062 (0.060)
Wealth third tertile	0.316** (0.073)	0.320** (0.073)	0.314** (0.073)	−0.122† (0.071)	−0.130† (0.071)	−0.120† (0.071)	−0.114 (0.071)	−0.120† (0.071)	−0.111 (0.071)	0.127† (0.074)	0.132† (0.074)	0.128† (0.074)
Married	0.125* (0.063)	0.116† (0.063)	0.120† (0.063)	−0.170** (0.062)	−0.153* (0.062)	−0.158* (0.061)	−0.166** (0.061)	−0.149* (0.061)	−0.154* (0.061)	0.145* (0.064)	0.136* (0.064)	0.138* (0.064)
Balaka	0.159* (0.065)	0.166* (0.064)	0.157* (0.065)	−0.185** (0.064)	−0.192** (0.063)	−0.184** (0.063)	−0.002 (0.063)	−0.009 (0.063)	0.000 (0.063)	−0.128† (0.066)	−0.116† (0.065)	−0.128† (0.066)
Rumphi	−0.010 (0.066)	0.002 (0.066)	−0.006 (0.066)	−0.100 (0.065)	−0.115† (0.065)	−0.105 (0.064)	0.069 (0.064)	0.053 (0.064)	0.063 (0.064)	−0.263** (0.067)	−0.254** (0.067)	−0.262** (0.067)
Constant	0.036 (0.094)	0.020 (0.094)	0.030 (0.094)	−0.074 (0.092)	−0.050 (0.092)	−0.067 (0.091)	−0.232* (0.091)	−0.226* (0.091)	−0.226* (0.091)	0.226* (0.095)	0.212* (0.095)	0.221* (0.095)
Observations	1,570	1,567	1,567	1,575	1,572	1,572	1,575	1,572	1,572	1,575	1,572	1,572

*Notes:* The table shows coefficients from linear regression models. The dependent variables are continuous measures. SF-12 mental is the 12-item short-form survey to provide a measure of mental health. Depression is measured with the PHQ-9 instrument that ranges from 0 to 27. Anxiety is measured with the GAD-7 instrument that ranges from 0 to 21. Finally, subjective well-being is measured with a 5-point Likert scale on life satisfaction. The reference categories are female, age <50 years, first tertile of the wealth distribution, single marital status (includes single, divorced, and widowed), and residing in Mchinji.

†*p* < .1, *p < .05, **p < .01.

Pain is also positively associated with standard definitions for the presence of at least mild and moderate depressive symptoms (Table 4). The relationship is again monotonic in pain severity and pain duration. Moderate depression is associated with at least moderate pain but is not associated with slight pain. Individuals with episodes of pain that lasted more than 3 months are 35% more likely to experience at least mild depression and 16% more likely to experience at least moderate depression. We also find a positive association between pain and having suicidal or self-harming thoughts. The association is particularly strong among those experiencing disabling pain or pain that lasted more than 3 months.

**Table 4.** Models Showing Association of Depression and Suicidal or Self-Harming Thoughts With Pain Severity and Duration

	At least mild depression			At least moderate depression			Suicidal or self-harming thoughts		
Regressors	(1)	(2)	(3)	(4)	(5)	(6)	(7)	(8)	(9)
Slight	0.081* (0.038)		0.080* (0.038)	0.019 (0.026)		0.018 (0.026)	0.034 (0.022)		0.034 (0.022)
Moderate	0.169** (0.032)		0.163** (0.033)	0.053* (0.022)		0.043* (0.022)	0.053** (0.019)		0.053** (0.019)
Severe	0.207** (0.039)		0.192** (0.041)	0.076** (0.025)		0.056* (0.026)	0.047* (0.022)		0.047* (0.023)
Disabling	0.215** (0.031)		0.198** (0.033)	0.093** (0.020)		0.076** (0.021)	0.070** (0.018)		0.066** (0.019)
Duration <1month		0.159** (0.025)			0.051** (0.017)			0.053** (0.015)	
Duration 2–3 months		0.204** (0.050)	0.017 (0.051)		0.098** (0.029)	0.036 (0.028)		0.045 (0.028)	−0.012 (0.027)
Duration >3 months		0.346** (0.069)	0.155* (0.070)		0.158** (0.035)	0.090** (0.034)		0.101** (0.031)	0.040 (0.030)
Male	−0.073** (0.028)	−0.075** (0.028)	−0.073** (0.028)	−0.005 (0.019)	−0.006 (0.018)	−0.004 (0.018)	−0.036* (0.016)	−0.037* (0.016)	−0.036* (0.016)
Age 50–59	0.018 (0.031)	0.012 (0.031)	0.015 (0.031)	−0.035† (0.019)	−0.038* (0.019)	−0.036† (0.019)	−0.044* (0.017)	−0.046** (0.017)	−0.045** (0.017)
Age 60–69	0.125** (0.035)	0.121** (0.035)	0.123** (0.035)	0.030 (0.023)	0.027 (0.023)	0.027 (0.023)	−0.009 (0.020)	−0.010 (0.021)	−0.009 (0.021)
Age 70+	0.237** (0.039)	0.228** (0.039)	0.230** (0.039)	0.059* (0.027)	0.049† (0.027)	0.050† (0.027)	0.013 (0.024)	0.010 (0.024)	0.011 (0.024)
Years of schooling	0.003 (0.005)	0.004 (0.005)	0.003 (0.005)	0.001 (0.003)	0.001 (0.003)	0.001 (0.003)	−0.004 (0.003)	−0.004 (0.003)	−0.004 (0.003)
Wealth second terile	−0.049† (0.029)	−0.058* (0.029)	−0.051† (0.029)	−0.035† (0.018)	−0.039* (0.018)	−0.038* (0.018)	−0.024 (0.015)	−0.024 (0.015)	−0.023 (0.015)
Wealth third tertile	−0.073* (0.035)	−0.075* (0.035)	−0.072* (0.035)	−0.037 (0.023)	−0.035 (0.023)	−0.034 (0.022)	0.008 (0.019)	0.009 (0.019)	0.009 (0.019)
Married	−0.076* (0.030)	−0.071* (0.030)	−0.073* (0.030)	−0.060** (0.019)	−0.054** (0.019)	−0.055** (0.018)	−0.022 (0.016)	−0.020 (0.016)	−0.021 (0.016)
Balaka	−0.107** (0.031)	−0.110** (0.031)	−0.105** (0.031)	−0.033† (0.019)	−0.037† (0.019)	−0.034† (0.019)	−0.037* (0.017)	−0.039* (0.016)	−0.036* (0.017)
Rumphi	−0.043 (0.032)	−0.048 (0.032)	−0.043 (0.032)	−0.008 (0.022)	−0.012 (0.022)	−0.010 (0.022)	−0.024 (0.019)	−0.024 (0.019)	−0.022 (0.019)
Observations	1575	1572	1572	1575	1572	1572	1575	1572	1572

*Notes:* The table shows average marginal effects from probit regressions. Dependent variables are based on the PHQ-9 instrument. At least mild depression refers to a PHQ-9 score of 5 or more while at least moderate depression to a score of 10 or more. Self-harming or suicidal thoughts refer to a response other than “not at all” to the final PHQ-9 question about suicide and self-harm. The reference categories are female, age <50 years, first tertile of the wealth distribution, single marital status (includes single, divorced, and widowed), and residing in Mchinji.

†*p* < .1, *p < .05, **p < .01.

## Discussion

We investigated the prevalence, severity, and duration of pain and its association with mental health and well-being in a population-based sample of 1,577 older adults aged 45 and older from three rural districts in Malawi. To our best knowledge, the association of pain with mental well-being among older individuals has not been previously documented in low-income contexts in SSA. Two key findings emerge from our analyses:

*Pain is highly prevalent among mature and older adults in rural Malawi*. Our analyses document a widespread prevalence of pain in this rural population with an average age of 60 years: 62% of the MLSFH-MAC study sample reported the experience of at least minor pain during the year before they were surveyed, and about one third reported severe or disabling pain. Moreover, while the prevalence of pain strongly increases with age at “young older ages,” the age gradient is modest older than age 60. Pain in this SSA LIC population is common and often severe across a substantial part of the life course at older ages.

Prior studies of pain in SSA LICs have mostly focused on prime-aged adults rather than mature and older adults. Importantly, our analyses show that the prevalence of pain is significantly higher among mature and older adults in Malawi than among younger study populations that were included in prior studies of pain in LICs (Stubbs et al., 2016). Moreover, the prevalence of pain in our sample also appears to be significantly higher than among older populations in high-income countries, despite the recent increases in pain among younger cohorts in the developed world (Zajkova et al., 2021; Zimmer et al., 2020). For example, the prevalence of any lasting pain among MLSFH mature adults is 40%–50% higher than among 10-year older participants in the U.S. HRS (Supplementary Figure 1; see Author Note 4).

Documenting this high prevalence of often severe pain among mature adults in SSA LIC is of substantial public health relevance because pain is frequently accompanied by impaired ability to perform central activities of daily living. Pain can also result in disability and physical limitations among older individuals (Andrews et al., 2013; Dueñas et al., 2020; Yiengprugsawan et al., 2017) and, therefore, sometimes pain can be interpreted as a sign of increasing frailty or accelerated aging.

In our study population, the most severe episodes of pain are generally reported to last 3 months or less, where 3 months is the standard cutoff for chronic pain (Treede et al., 2015). However, our findings do not rule out that older adults in rural Malawi may nonetheless suffer from chronic pain, as it is possible to experience multiple sequential episodes of pain in a year, but our study collected information only on the most severe pain episode during the reporting period.

Consistent with prior studies (Bingefors & Isacson, 2004; Fillingim et al., 2009), we found that—compared to men—women are more likely to report the experience of any pain and more severe pain. Although our data do not allow us to identify specific factors for these gender differences in pain, possible explanations include different biological and acquired risk factors, differences in reporting behaviors, differential access to health care and treatment, and differences in mortality (Bingefors & Isacson, 2004; Case & Paxson, 2005; Filingim et al., 2009).

*Pain is a strong predictor of poor mental health and subjective well-being among older adults in rural Malawi*. Our data show a strong negative association between pain and mental health and/or subjective well-being among mature and older adults. Longer and more severe pain episodes are associated with worse mental health and subjective well-being outcomes. These results are consistent across different measures of mental health including clinically validated measures of depression, anxiety, and indicators of subjective well-being. Our results show a strong gradient in the association of pain with the level of depression: Individuals reporting the experience of physical pain are also more likely to suffer from at least moderate depression and to entertain self-harming thoughts. Similar patterns have been documented among younger populations in LICs (Stubbs et al., 2016).

These associations between pain and mental health measures highlight the potentially significant contribution that widespread pain has on the well-being of mature and older adults in SSA LICs. Several recent studies have highlighted the rising burden of disease stemming from mental health in LICs (Patel 2007; Patel et al., 2018; Prince et al., 2007), including among older persons, and our findings point to the possibility that pain—as a broad indicator of declining physical health—is a likely key contributor to high levels of depression, anxiety, or poor subjective well-being among mature and older adults. While most individuals reporting pain also report taking some treatment (traditional and/or modern pain medicine), the strong associations of pain with mental health and/or well-being also indicate that these treatments may not necessarily be effective. While our analyses do not establish causation, these findings nevertheless indicate that prevention of pain and improving the treatment of widespread pain can be an important aspect in addressing the rising burden of depression, anxiety, and poor mental health in SSA LICs.

### Strengths and Limitations

Our study is based on a large, population-based sample of adults aged 45 years and older living in a rural SSA LIC context. Although this is not a nationally representative sample, the MLSFH-MAC cohort characteristics, prior comparisons with national samples, and the fact that roughly 85% of the Malawian population resides in rural areas with similar conditions provide reassurance that our findings are locally valid and can be potentially generalized to other rural areas in Malawi and similar low-income populations in southeastern SSA. The age range covered in our study (age 45+ years) is comparable to other aging studies in SSA LICs (Brinkmann et al., 2020), and given life expectancy trends in Malawi and SSA more generally, the study represents adequately the experience of the older population in the region (United Nations Department of Economic and Social Affairs, & Population Division, 2019).

MLSFH-MAC is one of the rare cohort studies in a SSA LICs that provides detailed information on multiple dimensions of mental health and well-being covered in this analysis. Specifically, our measures of mental health are based on clinically validated instruments such as PHQ-9 and GAD-7, the SF-12 mental health score, and indicators of overall subjective well-being. To our best knowledge, no study investigating the association of pain with mental health has utilized such a comprehensive set of measures. Although our findings pertain to individuals aged 45 and older and thus no information about the prevalence of pain at younger ages can be derived from our study, our findings are of particular importance because prior research in this context has primarily documented patterns of pain among younger individuals while neglecting older people who are the most likely to experience pain.

Some limitations of this study are noteworthy. The MLSFH-MAC data lack details on the type of pain experienced by the study participants. Our work is therefore silent on what type of pain is more prevalent in this population and has little to say on how individuals cope with the experience of pain. Likewise, MLSFH-MAC does not include information on pain loci, which itself is potentially relevant to better understand the gender differences in pain documented in our study. For instance, prior research has widely documented that women are more likely to experience chronic pain (Berkley, 1997; Institute of Medicine 2001) as a result from the combination of physiological, genetic, and environmental factors. While a number of chronic pain conditions occur only among women (e.g., menstrual pain, endometriosis), the chronic pain syndromes with the highest overall prevalence are also more likely to be reported by women (e.g., low back pain, neck pain, migraine, and headaches; Mogil, 2012). Moreover, gender differences in the perception of pain may also contribute to these patterns.

Similarly, we have only limited information on respondents’ pain management strategies, and data on the type or duration of pain treatment are not available. To investigate other potential coping strategies to alleviate pain in this context, we investigated the use of alcohol as an analgesic, but were not able to find a statistically significant relationship between pain and alcohol consumption.

The present analysis is cross-sectional and focuses on the basic relationship between pain and mental well-being in a low-income SSA population. It is important to acknowledge that our analysis does not establish causation. More research is also needed to understand how patterns of pain change over time, and how these changes relate to longitudinal trajectories and outcomes of mental health. For instance, only 37% of respondents reported the experience of any pain in 2017 and 2018, while about 25% of MLSFH-MAC respondents who reported pain in 2017 did not do so a year later; vice versa, 15% of respondents reported having pain in 2018 but not in 2017. Conditional on experiencing pain in 2017, however, about 60% of study participants also report pain in 2018. These numbers suggest that the experience of pain is dynamic over time, and they further reinforce our conclusion that pain is also often persistent for a substantial fraction of individuals. Additional dynamic analyses controlling for individual fixed effects, and possibly also incorporating additional aspects such as distance and access to health providers, will help to better understand the relationship between pain and mental well-being over time. These analyses are beyond the scope of the current article.

Like any other longitudinal survey, the original MLSFH sample has been affected by mortality and other attrition. Because the MLSFH-MAC sample utilized in this analysis is the evolution of the original 1998 probability sample through additional enrollments and attrition, there are currently no sampling weights. Therefore, it is possible that our findings may not fully generalize to the rural Malawi population aged 45 and older as the MLSFH-MAC is not a fully representative sample of this population.

### Implications of Our Findings for Public Health Policy in SSA LICs

Our study has important implications for public health policy in SSA LICs such as Malawi. The staggering prevalence of pain among older adults in Malawi calls for policy interventions and welfare support. This is particularly important in this context because older adults rely to a large extent on strenuous physical work for their sustenance (Payne et al., 2017). The strong association with severe forms of depression also calls for targeted mental health interventions, particularly among women. Kohler et al. (2017) have shown that depression and anxiety alone are associated with adverse outcomes, such as poorer nutrition intake and reduced work effort. The combination of poor mental health and experience of pain suggests even a higher burden of diseases and a higher adverse impact on individual well-being.

Finally, one concern for public health policy emerging from these findings is that while a substantial fraction of our respondents report receiving some treatment for pain (only 5.4% of MLSFH-MAC study participants did not use any treatment for their most severe episode of pain during the past year lasting 1 week or longer; Panel C, Table 1), the high prevalence and severity of pain suggest that the treatment used by older adults is not effective in addressing their medical needs. Future work should investigate the type of pain treatment provided in LICs and propose ways to improve the response of the health care systems to this major health problem. In this low-income setting where care for older adults is largely informal (Aboderin, 2010; Kohler et al., 2012), ineffective pain treatments point toward a substantial burden on family members who are unlikely to address the health care needs of a growing older population experiencing disabling conditions such as pain or poor mental health.

## Conclusion

Our study is one of the first studies that assess the sociodemographic distribution of pain in a nonclinical SSA LIC’s population and investigates its relevance as a predictor for subjective well-being and mental health among older adults utilizing population-based data. Our findings are of particular public health relevance because they contribute to a better understanding of the needs of older people in SSA resource-constraint settings and help identify key subpopulations that are particularly affected and limited by the experience of pain in their daily life. Our results emphasize the importance of prioritizing chronic conditions among the older individuals in SSA LICs, to which relatively few health care resources continue to be allocated.

## Supplementary Material

igac008_suppl_Supplementary_Material

## Author Notes

1. Differences arise in the distribution of religion, where Muslims are overrepresented in MLSFH-MAC because about one third of the MLSFH-MAC study population is from the primarily Muslim region of Balaka. Individuals aged 65 and older in the MLSFH-MAC were somewhat more likely to have ever attended school than those in the IHS3 national sample. The MLSFH-MAC also contains a larger fraction of female respondents and of respondents who are currently married, both of which are likely due to the initial 1998 MLSFH sample that focused on ever-married women and their spouses and from which members of the aging cohort were recruited.

2. As expected, given the high mobility of the rural population, attrition in the MLSFH is nontrivial and predicted by several observable characteristics (such as age, gender, HIV status). The primary cause for loss to follow-up in the MLSFH-MAC is mortality. Excluding deceased respondents the MLSFH-MAC successfully surveyed in 2018, the currently last follow-up of the cohort, a remarkable 97% of the respondents interviewed at baseline in 2012, providing a very high rate of retention of study participants in this mature cohort. Tests for selective attrition in the MLSFH-MAC indicate that loss to follow-up (due to attrition or mortality) does not necessarily bias the coefficients of estimated multivariate relationships that control for key socioeconomic characteristics (Kohler et al., 2015, 2020).

3. The total number of respondents with missing information on pain, age, gender, schooling, and wealth was 33, or 2% of the entire sample. Therefore, it is very unlikely that omitting these observations with missing values from the analysis had an impact on the results.

4. We compared MLSFH-MAC respondents with HRS respondents 10 years older because life expectancy in Malawi at age 45 is equal to the life expectancy in the United States at age 55 (around 27 years in both cases during 2015–2020; United Nations Development Programme, 2019). An additional rationale for comparing MLSFH-MAC respondents to older HRS respondents is that the HRS does not include individuals younger than 50 years.
