# Supplementary material for: Pain Is Widespread and Predicts Poor Mental Health Among Older Adults in Rural Malawi
Source: Innov Aging. 2022 Mar 5;6(3):igac008. doi: 10.1093/geroni/igac008 (PMC9074811; doi:10.1093/geroni/igac008)
Supplement: igac008_suppl_Supplementary_Material [file igac008_suppl_supplementary_material.pdf]

Supplementary material for "Pain is Widespread and Predicts Poor Mental Health and Subjective Wellbeing among Older Adults in Rural Malawi"

Table S1: Models Showing Association Between Mental Health and Wellbeing with Pain Severity and Duration: Women

	SF-12 mental			depression			anxiety			wellbeing		
	(1)	(2)	(3)	(4)	(5)	(6)	(7)	(8)	(9)	(10)	(11)	(12)
**slight**	-0.135		-0.133	0.131		0.130	0.168+		0.166+	-0.096		-0.096
	(0.104)		(0.103)	(0.101)		(0.100)	(0.101)		(0.100)	(0.099)		(0.099)
**moderate**	-0.221*		-0.192*	0.339**		0.299**	0.363**		0.330**	-0.192*		-0.193*
	(0.096)		(0.096)	(0.093)		(0.093)	(0.093)		(0.093)	(0.091)		(0.092)
**severe**	-0.251*		-0.123	0.555**		0.450**	0.584**		0.488**	-0.294**		-0.299**
	(0.112)		(0.116)	(0.108)		(0.113)	(0.108)		(0.113)	(0.106)		(0.111)
**disabling**	-0.400**		-0.320**	0.583**		0.497**	0.623**		0.554**	-0.187*		-0.182*
	(0.088)		(0.091)	(0.086)		(0.089)	(0.086)		(0.088)	(0.084)		(0.087)
**age 50-59**	-0.132	-0.131	-0.135	0.042	0.038	0.041	0.050	0.045	0.049	-0.167*	-0.164*	-0.162*
	(0.087)	(0.086)	(0.086)	(0.084)	(0.084)	(0.084)	(0.084)	(0.084)	(0.083)	(0.082)	(0.082)	(0.082)
**age 60-69**	-0.208*	-0.198*	-0.199*	0.241*	0.219*	0.227*	0.267**	0.247**	0.256**	-0.141	-0.138	-0.143
	(0.099)	(0.098)	(0.098)	(0.096)	(0.096)	(0.095)	(0.096)	(0.096)	(0.095)	(0.094)	(0.094)	(0.094)
**age 70+**	-0.351**	-0.305**	-0.311**	0.521**	0.474**	0.481**	0.632**	0.587**	0.594**	-0.415**	-0.400**	-0.400**
	(0.112)	(0.112)	(0.112)	(0.109)	(0.109)	(0.109)	(0.109)	(0.109)	(0.109)	(0.107)	(0.107)	(0.107)
**Years of schooling**	-0.008	-0.008	-0.006	0.012	0.012	0.009	0.015	0.016	0.012	-0.000	0.001	0.002
	(0.015)	(0.015)	(0.015)	(0.015)	(0.015)	(0.015)	(0.015)	(0.015)	(0.015)	(0.014)	(0.014)	(0.014)
**wealth 2nd ter**	0.188*	0.205**	0.202**	-0.099	-0.137+	-0.117	0.002	-0.034	-0.014	0.031	0.048	0.037

	(0.076)	(0.076)	(0.076)	(0.074)	(0.074)	(0.074)	(0.074)	(0.074)	(0.074)	(0.073)	(0.072)	(0.073)
**wealth 3rd ter**	0.330**	0.321**	0.327**	-0.136	-0.140	-0.136	-0.067	-0.069	-0.065	0.087	0.096	0.090
	(0.105)	(0.104)	(0.104)	(0.102)	(0.101)	(0.101)	(0.101)	(0.101)	(0.101)	(0.100)	(0.099)	(0.099)
**married**	0.153*	0.149*	0.150*	-0.201**	-0.182*	-0.187**	-0.231**	-0.215**	-0.220**	0.180*	0.168*	0.170*
	(0.075)	(0.074)	(0.074)	(0.073)	(0.073)	(0.072)	(0.072)	(0.073)	(0.072)	(0.071)	(0.071)	(0.071)
**Balaka**	0.157+	0.171*	0.157+	-0.262**	-0.277**	-0.262**	-0.031	-0.047	-0.031	-0.082	-0.082	-0.083
	(0.085)	(0.084)	(0.085)	(0.083)	(0.082)	(0.082)	(0.083)	(0.082)	(0.082)	(0.081)	(0.080)	(0.081)
**Rumphi**	-0.005	0.006	-0.001	-0.157+	-0.172+	-0.160+	0.021	0.002	0.015	-0.215*	-0.212*	-0.214*
	(0.094)	(0.094)	(0.094)	(0.091)	(0.091)	(0.091)	(0.091)	(0.091)	(0.091)	(0.090)	(0.089)	(0.089)
**duration < 1 month**		-0.210**			0.344**			0.386**			-0.181**	
		(0.071)			(0.069)			(0.069)			(0.068)	
**duration 2-3 months**		-0.627**	-0.409**		0.685**	0.241+		0.703**	0.214		-0.041	0.190
		(0.145)	(0.144)		(0.141)	(0.140)		(0.141)	(0.140)		(0.138)	(0.137)
**duration > 3 months**		-0.623**	-0.361*		1.054**	0.593**		0.969**	0.459**		-0.451**	-0.242
		(0.180)	(0.180)		(0.175)	(0.174)		(0.175)	(0.174)		(0.172)	(0.172)
**Constant**	-0.027	-0.047	-0.044	-0.047	-0.019	-0.029	-0.267*	-0.238*	-0.248*	0.187	0.175	0.180
	(0.122)	(0.122)	(0.122)	(0.119)	(0.118)	(0.118)	(0.118)	(0.118)	(0.118)	(0.116)	(0.116)	(0.116)
**Observations**	948	946	946	949	947	947	949	947	947	949	947	947

Table S2: Models Showing Association Between Mental Health and Wellbeing with Pain Severity and Duration: Men

	**SF-12 mental**			**depression**			**anxiety**			**wellbeing**		
	(1)	(2)	(3)	(4)	(5)	(6)	(7)	(8)	(9)	(10)	(11)	(12)
**slight**	-0.160		-0.163	0.170		0.167	0.111		0.105	0.159		0.172
	(0.124)		(0.124)	(0.124)		(0.124)	(0.122)		(0.122)	(0.139)		(0.139)
**moderate**	-0.339**		-0.355**	0.304**		0.305**	0.220*		0.217*	-0.244*		-0.215+
	(0.099)		(0.100)	(0.098)		(0.099)	(0.097)		(0.098)	(0.110)		(0.112)
**severe**	-0.594**		-0.603**	0.460**		0.438**	0.332**		0.287*	-0.036		0.037
	(0.127)		(0.131)	(0.125)		(0.129)	(0.123)		(0.127)	(0.140)		(0.145)
**disabling**	-0.469**		-0.470**	0.447**		0.418**	0.381**		0.327**	-0.212+		-0.134
	(0.108)		(0.113)	(0.108)		(0.113)	(0.106)		(0.111)	(0.121)		(0.126)
**age 50-59**	0.037	0.077	0.048	-0.047	-0.081	-0.061	-0.015	-0.051	-0.037	-0.183	-0.167	-0.165
	(0.102)	(0.102)	(0.102)	(0.101)	(0.101)	(0.101)	(0.099)	(0.099)	(0.099)	(0.113)	(0.113)	(0.114)
**age 60-69**	-0.211*	-0.201+	-0.207*	0.226*	0.218*	0.222*	0.228*	0.220*	0.221*	-0.215+	-0.221+	-0.210+
	(0.103)	(0.103)	(0.103)	(0.102)	(0.102)	(0.102)	(0.100)	(0.100)	(0.100)	(0.115)	(0.115)	(0.115)
**age 70+**	-0.417**	-0.400**	-0.411**	0.562**	0.542**	0.550**	0.611**	0.586**	0.590**	-0.553**	-0.520**	-0.525**
	(0.110)	(0.111)	(0.110)	(0.109)	(0.109)	(0.110)	(0.107)	(0.107)	(0.108)	(0.122)	(0.123)	(0.123)
**years of schooling**	-0.018	-0.020	-0.018	0.006	0.008	0.007	0.003	0.005	0.004	-0.000	-0.004	-0.002
	(0.013)	(0.013)	(0.013)	(0.013)	(0.013)	(0.013)	(0.013)	(0.013)	(0.013)	(0.015)	(0.015)	(0.015)
**wealth 2nd ter**	0.136	0.141	0.122	-0.117	-0.124	-0.110	-0.058	-0.060	-0.049	0.106	0.126	0.111
	(0.098)	(0.098)	(0.098)	(0.097)	(0.098)	(0.098)	(0.096)	(0.096)	(0.096)	(0.110)	(0.110)	(0.110)

**wealth 3rd ter**	0.294**	0.287**	0.276**	-0.103	-0.102	-0.093	-0.170+	-0.165+	-0.156	0.165	0.186	0.171
	(0.102)	(0.102)	(0.102)	(0.101)	(0.102)	(0.102)	(0.100)	(0.100)	(0.100)	(0.114)	(0.114)	(0.114)
**married**	0.043	0.072	0.058	-0.063	-0.080	-0.073	0.124	0.107	0.110	0.084	0.085	0.088
	(0.146)	(0.147)	(0.147)	(0.146)	(0.146)	(0.146)	(0.144)	(0.144)	(0.144)	(0.164)	(0.165)	(0.164)
**Balaka**	0.163	0.134	0.146	-0.059	-0.049	-0.051	0.031	0.040	0.043	-0.184	-0.139	-0.176
	(0.102)	(0.102)	(0.103)	(0.102)	(0.101)	(0.102)	(0.100)	(0.099)	(0.101)	(0.115)	(0.114)	(0.115)
**Rumphi**	-0.042	-0.040	-0.055	-0.048	-0.053	-0.042	0.090	0.087	0.096	-0.327**	-0.295**	-0.320**
	(0.093)	(0.093)	(0.093)	(0.092)	(0.092)	(0.093)	(0.091)	(0.091)	(0.091)	(0.104)	(0.104)	(0.104)
**duration < 1 month**		-0.382**			0.326**			0.232**			-0.071	
		(0.077)			(0.076)			(0.075)			(0.086)	
**duration 2-3 months**		-0.308*	0.154		0.390*	0.010		0.343*	0.068		-0.364*	-0.279
		(0.155)	(0.156)		(0.154)	(0.155)		(0.151)	(0.153)		(0.173)	(0.175)
**duration > 3 months**		-0.794**	-0.325		0.749**	0.357		0.848**	0.560*		-0.512+	-0.428
		(0.243)	(0.242)		(0.241)	(0.242)		(0.237)	(0.238)		(0.272)	(0.272)
**Constant**	0.356+	0.328+	0.359+	-0.365+	-0.341+	-0.363+	-0.595**	-0.576**	-0.591**	0.394+	0.366+	0.379+
	(0.191)	(0.192)	(0.191)	(0.190)	(0.190)	(0.191)	(0.188)	(0.187)	(0.187)	(0.214)	(0.214)	(0.214)
**Observations**	622	621	621	626	625	625	626	625	625	626	625	625

Table S3: Models Showing Association of Depression with Pain Severity and Duration: Women

	mild depression			Moderate depression			Suicidal thoughts		
	(1)	(2)	(3)	(4)	(5)	(6)	(7)	(8)	(9)
**slight**	0.075		0.074	0.007		0.007	0.047		0.047
	(0.049)		(0.049)	(0.035)		(0.035)	(0.031)		(0.031)
**moderate**	0.189**		0.176**	0.033		0.018	0.065*		0.061*
	(0.044)		(0.044)	(0.031)		(0.031)	(0.028)		(0.028)
**severe**	0.238**		0.206**	0.089**		0.064+	0.036		0.025
	(0.051)		(0.054)	(0.033)		(0.035)	(0.034)		(0.035)
**disabling**	0.234**		0.209**	0.098**		0.080**	0.102**		0.089**
	(0.040)		(0.042)	(0.027)		(0.027)	(0.025)		(0.026)
**age 50-59**	0.033	0.033	0.033	-0.034	-0.036	-0.034	-0.050*	-0.053*	-0.051*
	(0.041)	(0.041)	(0.041)	(0.026)	(0.026)	(0.026)	(0.024)	(0.025)	(0.025)
**age 60-69**	0.126**	0.118*	0.122**	0.022	0.017	0.018	-0.004	-0.008	-0.006
	(0.047)	(0.047)	(0.047)	(0.032)	(0.032)	(0.032)	(0.030)	(0.030)	(0.030)
**age 70+**	0.192**	0.179**	0.181**	0.070+	0.055	0.059	0.028	0.020	0.024
	(0.054)	(0.054)	(0.054)	(0.040)	(0.039)	(0.039)	(0.037)	(0.037)	(0.037)
**years of schooling**	0.006	0.006	0.006	0.004	0.003	0.003	-0.010*	-0.010*	-0.010*
	(0.007)	(0.007)	(0.007)	(0.005)	(0.005)	(0.005)	(0.005)	(0.005)	(0.005)
**wealth 2nd ter**	-0.071*	-0.084*	-0.076*	-0.035	-0.042+	-0.041+	-0.043*	-0.045*	-0.046*

	(0.036)	(0.036)	(0.036)	(0.023)	(0.023)	(0.023)	(0.021)	(0.021)	(0.021)
**wealth 3rd ter**	-0.103*	-0.103*	-0.102*	-0.052	-0.049	-0.050	0.003	0.006	0.003
	(0.049)	(0.049)	(0.049)	(0.034)	(0.033)	(0.033)	(0.029)	(0.029)	(0.029)
**married**	-0.084*	-0.079*	-0.082*	-0.069**	-0.062**	-0.062**	-0.009	-0.008	-0.007
	(0.035)	(0.035)	(0.035)	(0.023)	(0.023)	(0.023)	(0.022)	(0.022)	(0.022)
**Balaka**	-0.150**	-0.158**	-0.151**	-0.058*	-0.060*	-0.058*	-0.042+	-0.047*	-0.042+
	(0.040)	(0.039)	(0.040)	(0.026)	(0.026)	(0.026)	(0.023)	(0.023)	(0.023)
**Rumphi**	-0.080+	-0.085+	-0.081+	-0.032	-0.037	-0.034	-0.008	-0.009	-0.006
	(0.045)	(0.045)	(0.045)	(0.031)	(0.031)	(0.031)	(0.030)	(0.030)	(0.030)
**duration < 1month**		0.167**			0.046*			0.064**	
		(0.033)			(0.023)			(0.023)	
**duration 2-3 months**		0.300**	0.096		0.100*	0.036		0.088*	0.026
		(0.068)	(0.068)		(0.040)	(0.038)		(0.038)	(0.036)
**duration > 3months**		0.358**	0.151+		0.173**	0.104*		0.146**	0.070+
		(0.087)	(0.088)		(0.045)	(0.043)		(0.042)	(0.040)
**Observations**	949	947	947	949	947	947	949	947	947

Table S4: Models Showing Association of Depression with Pain Severity and Duration: Men

	mild depression			moderate depression			suicidal thoughts		
	(1)	(2)	(3)	(4)	(5)	(6)	(7)	(8)	(9)
**slight**	0.113+		0.114+	0.040		0.038	0.018		0.021
	(0.061)		(0.061)	(0.040)		(0.039)	(0.031)		(0.032)
**moderate**	0.154**		0.163**	0.075*		0.073*	0.035		0.040+
	(0.048)		(0.048)	(0.030)		(0.030)	(0.022)		(0.023)
**severe**	0.157*		0.164**	0.051		0.039	0.056*		0.069*
	(0.061)		(0.063)	(0.038)		(0.039)	(0.025)		(0.028)
**disabling**	0.186**		0.186**	0.079*		0.066*	0.023		0.032
	(0.051)		(0.053)	(0.032)		(0.033)	(0.025)		(0.027)
**age 50-59**	-0.015	-0.025	-0.022	-0.038	-0.041	-0.042	-0.032	-0.038	-0.035
	(0.047)	(0.047)	(0.047)	(0.027)	(0.027)	(0.027)	(0.022)	(0.024)	(0.024)
**age 60-69**	0.120*	0.118*	0.117*	0.038	0.040	0.037	-0.010	-0.016	-0.012
	(0.051)	(0.051)	(0.051)	(0.033)	(0.034)	(0.034)	(0.025)	(0.027)	(0.026)
**age 70+**	0.284**	0.280**	0.281**	0.043	0.038	0.038	-0.001	-0.001	-0.000
	(0.056)	(0.057)	(0.057)	(0.036)	(0.036)	(0.037)	(0.027)	(0.030)	(0.029)
**years of schooling**	0.001	0.002	0.001	-0.001	-0.001	-0.001	0.001	0.001	0.001
	(0.007)	(0.007)	(0.007)	(0.004)	(0.004)	(0.004)	(0.003)	(0.003)	(0.003)
**wealth 2nd ter**	-0.006	-0.001	0.002	-0.032	-0.030	-0.031	0.011	0.015	0.017
	(0.049)	(0.048)	(0.048)	(0.029)	(0.029)	(0.029)	(0.022)	(0.023)	(0.023)

**wealth 3rd ter**	-0.028	-0.021	-0.018	-0.018	-0.015	-0.015	0.013	0.018	0.017
	(0.050)	(0.050)	(0.051)	(0.030)	(0.030)	(0.030)	(0.023)	(0.024)	(0.024)
**married**	-0.119+	-0.127+	-0.126+	-0.037	-0.037	-0.038	-0.041	-0.050+	-0.048+
	(0.069)	(0.068)	(0.068)	(0.040)	(0.040)	(0.040)	(0.026)	(0.028)	(0.028)
**Balaka**	-0.031	-0.023	-0.019	0.003	-0.002	0.003	-0.021	-0.015	-0.019
	(0.050)	(0.050)	(0.050)	(0.029)	(0.029)	(0.029)	(0.023)	(0.024)	(0.024)
**Rumphi**	-0.002	0.004	0.007	0.017	0.015	0.018	-0.024	-0.024	-0.022
	(0.047)	(0.046)	(0.047)	(0.030)	(0.030)	(0.030)	(0.020)	(0.021)	(0.021)
**duration < 1month**		0.159**			0.059*			0.042*	
		(0.037)			(0.025)			(0.019)	
**duration 2-3 months**		0.073	-0.097		0.092*	0.033		0.000	0.000
		(0.076)	(0.077)		(0.042)	(0.041)		(.)	(.)
**duration > 3months**		0.357**	0.182		0.129*	0.071		0.054	0.007
		(0.115)	(0.116)		(0.059)	(0.058)		(0.051)	(0.050)
**Observations**	626	625	625	626	625	625	626	586	586

Figure S1: Comparison of prevalence of any pain between MLSFH mature adults and HRS respondents 10 years older

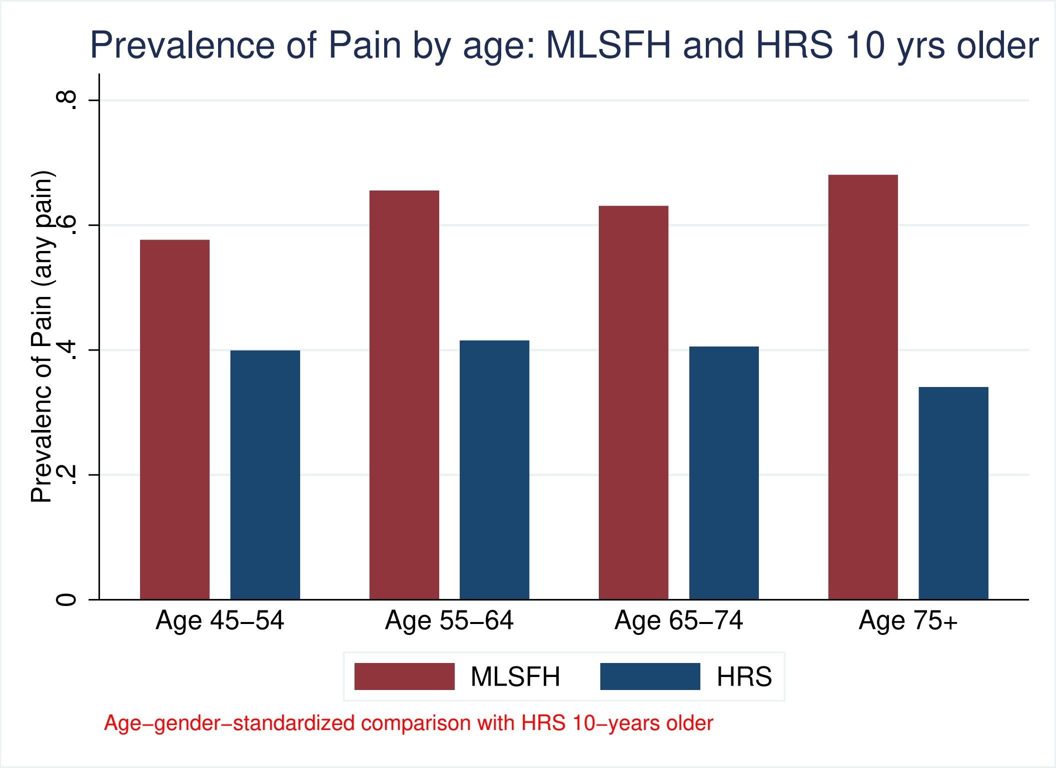

Notes: The figure reports the proportion or respondents in the MLSFH and 2010 Health and Retirement Survey (HRS) who reported having experienced any pain in response to the question "During the past year, have you experienced pain that lasted for one week or longer?" (this question was identically asked in the 2010 HRS pain module (Module 6) and the MLSFH. The HRS population is weighted to match the MLSFH age and gender distribution to eliminate differences rated to different age or gender (also within each age group). The bars show the prevalence of any pain between MLSFH respondents as compared to HRS respondents 10 years older.
